# Improving the Voltammetric Determination of Hg(II): A Comparison Between Ligand-Modified Glassy Carbon and Electrochemically Reduced Graphene Oxide Electrodes

**DOI:** 10.3390/s20236799

**Published:** 2020-11-28

**Authors:** Matei D. Raicopol, Andreea M. Pandele, Constanţa Dascălu, Eugeniu Vasile, Anamaria Hanganu, Gabriela-Geanina Vasile, Ioana Georgiana Bugean, Cristian Pirvu, Gabriela Stanciu, George-Octavian Buica

**Affiliations:** 1 Faculty of Applied Chemistry and Materials Science, University Politehnica of Bucharest, 1-7 Gheorghe Polizu St., 011061 Bucharest, Romania; m_raicopol@chim.upb.ro (M.D.R.); madalina.pandele@upb.ro (A.M.P.); eugeniu.vasile@upb.ro (E.V.); ioana.g.lazar@gmail.com (I.G.B.); cristian.pirvu@upb.ro (C.P.); 2 Faculty of Applied Sciences, University Politehnica of Bucharest, 313 Splaiul Independenţei, 060042 Bucharest, Romania; constanta_dascalu@physics.pub.ro; 3 Department of Organic Chemistry, Biochemistry and Catalysis, University of Bucharest, 90-92 Sos. Panduri, 050657 Bucharest, Romania; anamaria.hanganu@unibuc.ro; 4 National Research and Development Institute for Industrial Ecology ECOIND Bucharest, 71-73 Drumul Podul Dambovitei Street, 060652 Bucharest, Romania; gabriela.vasile@incdecoind.ro; 5 Department of Chemistry and Chemical Engineering, Ovidius University, 124 Mamaia Blvd, 900527 Constanta, Romania

**Keywords:** modified electrodes, electrochemically reduced graphene oxide, thiosemicarbazone, click chemistry, anodic stripping voltammetry, mercury determination

## Abstract

A new thiosemicarbazone ligand was immobilized through a Cu(I)-catalyzed click reaction on the surface of glassy carbon (GC) and electrochemically reduced graphene oxide (GC-ERGO) electrodes grafted with phenylethynyl groups. Using the accumulation at open circuit followed by anodic stripping voltammetry, the modified electrodes showed a significant selectivity and sensibility for Hg(II) ions. A detection limit of 7 nM was achieved with the GC modified electrodes. Remarkably, GC-ERGO modified electrodes showed a significantly improved detection limit (0.8 nM), sensitivity, and linear range, which we attribute to an increased number of surface binding sites and better electron transfer properties. Both GC and GC-ERGO modified electrodes proved their applicability for the analysis of real water samples.

## 1. Introduction

Due to their negative impact on human health [1], the maximum permissible concentration of heavy metal ions (e.g., Pb^2+^, Cd^2+^, Hg^2+^, Ni^2+^) in environmental samples is in the part per billion (ppb) range, so their detection is a demanding issue. For example, according to European Union legislation, the maximum allowable concentration of inorganic mercury ions in drinking water is 1 ppb [2]. Both industrial activities [3,4] and climate change [5,6] can generate emissions of mercury into the environment, whether in water, soil, or air. 

Various analytical techniques are currently employed for the detection of Hg(II) in environmental samples: atomic fluorescence spectrometry [7,8,9], UV-VIS spectrophotometry [10], inductively coupled plasma optical emission [11,12] and mass spectrometry [13], and cold vapor generation-quartz crystal microbalance [14]. Hyphenated methods such as gas chromatography-tandem mass spectrometry [15] and high-performance liquid chromatography-inductively coupled plasma mass spectrometry [16] allow both detection and speciation of mercury compounds. Generally, these techniques require high operating costs, due to expensive equipment and sophisticated sample processing.

In this context, heavy metal analysis using electrochemical methods is becoming an alternative for more sophisticated and costly techniques [17]. Particularly suitable for the trace analysis of heavy metals is the voltammetric determination following preconcentration at electrodes modified with complexing agents, which enhances both selectivity and sensitivity [18]. 

A versatile method for electrode surface modification is through click reactions such as Cu(I)-catalyzed azide-alkyne cycloaddition [19], but to date there are few reports of its application for ligand-modified electrodes [20,21,22,23]. A shortcoming of this approach is that it requires substrates previously grafted with a layer containing azide or alkyne functionalities. Usually, this leads to an increase in charge transfer resistance that affects the analytical response of the modified electrode. One way to overcome this drawback is through the use of graphene substrates, which offer improved electron transfer properties [24,25,26,27]. 

Since it has recently been pointed out that reduced graphene oxide is beneficially utilized when the electrochemical detection mechanism is adsorptive in nature [28], as in the case of stripping voltammetry, in this work we employ glassy carbon (GC) and electrochemically reduced graphene oxide (ERGO) electrodes modified with a new thiosemicarbazone ligand for the stripping voltammetric analysis of Hg(II). 

Electrode modification is accomplished using the two-step functionalization protocol recently developed in our group [29], which involves surface grafting with phenylethynyl groups via the corresponding diazonium salt and the subsequent ligand immobilization through a Cu(I)-catalyzed azyde-alkyne cycloaddition. The extent of surface modification is assessed through various techniques, and we demonstrate that ERGO-based electrodes have an increased number of surface binding sites and better electron transfer properties that lead to a significant improvement in analytical performance. 

## 2. Materials and Methods

### 2.1. Reagents

N,N-dimethylformamide (DMF), acetonitrile (MeCN, electronic grade, 99.999%), absolute ethanol (EtOH), diethyl ether (Et_2_O), tetrabutylammonium tetrafluoroborate (TBABF_4_, electrochemical grade), ferrocenemethanol (FcMeOH), bromotris(triphenylphosphine) copper(I) (CuBr(PPh_3_)_3_), triethylamine (TEA) and graphene oxide were purchased from Sigma-Aldrich and used as received. Acetic acid, sodium acetate and metal salts (analytical reagent grade) were purchased from Sigma-Aldrich or Merck and used without further purification. 

4-(6-azidohexyl)-3-thiosemicarbazide and 4-ethynylphenyldiazonium tetrafluoroborate were synthesized as described in our previous paper [29].

All aqueous solutions were prepared using Type I ultrapure water (resistivity 18.2 MΩ·cm).

### 2.2. Equipment

NMR spectra were recorded on a Bruker Avance III 500 (^1^H: 500 MHz, ^13^C: 125 MHz) spectrometer. Infrared spectra were recorded in the 4000–600 cm^−1^ range, at a resolution of 4 cm^−1^, on a Bruker Vertex 70 FT-IR spectrometer. UV-Vis spectra were recorded in the 200–850 nm range using a Jasco V-670 double-beam spectrometer fitted with a standard 1 cm quartz cuvette. Raman spectra were obtained with a Renishaw InVia confocal Raman microscope fitted with a 473 nm excitation laser, at 0.4 mW incident power and a resolution of 2 cm^−1^. Scanning electron microscopy (SEM) investigations were carried out using a FEI Quanta Inspect F electron microscope.

X-ray photoelectron spectroscopy (XPS) analysis was performed on a Thermo Scientific K-Alpha spectrometer with a monochromated Al Kα source (1486.6 eV). A combined electron/argon ion flood source was employed for charge neutralization. Survey and high-resolution spectra were recorded at pass energies of 200 eV and 20 eV, respectively. Binding energies were referenced to the C 1s peak at 284.5 eV (graphitic carbon). Atomic concentrations were calculated using sensitivity factors supplied by the instrument manufacturer. 

A three-electrode electrochemical cell connected to a Metrohm Autolab PGSTAT204 potentiostat/galvanostat was used to perform the electrochemical experiments. Bare or modified glassy carbon (GC) disks (3 mm diameter, from ALS Co. Ltd. Tokyo, Japan) were used as working electrodes. A Pt wire served as counter electrode and Ag/10 mM Ag^+^, 0.1 M TBABF_4_ in DMF or Ag/AgCl (3 M KCl) reference electrodes were used for nonaqueous and aqueous solutions, respectively. A Metrohm Autolab PGSTAT302N potentiostat/galvanostat fitted with a FRA32M frequency response analyzer was employed for performing electrochemical impedance spectroscopy (EIS). Impedance spectra were recorded in the 100 kHz–10 mHz frequency range, with an AC signal amplitude of 10 mV and a DC bias potential of 0.25 V.

A Thermo Scientific M6 Dual atomic absorption spectrometer (AAS) with dual background correction and a VP 100 continuous flow vapor generator (VG) was used to quantify the Hg(II) amount in tap water samples. Alternatively, a Cetac Technologies Quick Trace M-8000 Mercury Analyzer was also used for Hg(II) quantification through cold vapor atomic fluorescence spectroscopy (CV-AFS).

### 2.3. Procedures

#### 2.3.1. Synthesis of Ligand **L**

To a solution of 0.7 g (4.7 mmol) phthalaldehydic acid in 5 mL absolute ethanol heated to reflux was added dropwise a solution of 1 g (4.7 mmol) 4-(6-azidohexyl)-3-thiosemicarbazide dissolved in 5 mL of absolute ethanol. The obtained mixture was refluxed for a period of 4 h and then cooled to room temperature. Using a rotary evaporator, about 2/3 of the ethanol was removed, and the residue was maintained at −20 °C for 24 h. The crystallized thiosemicarbazone was filtered under vacuum, and consecutively washed with cold ethanol, cold diethyl ether, and finally dried under vacuum (5 mmHg) at room temperature. Yield: 0.5 g (31%) as off-white crystals.

*Characterization of ligand **L***:

^1^H-NMR (500 MHz, DMSO-d_6_, ppm) δ: 1.30–1.39 (m, 4H); 1.53–1.62 (m, 4H); 3.31 (t, *J* = 6.9 Hz, 2H); 3.55 (quartet, *J* = 6.4 Hz, 2H); 7.48 (t, *J* = 7.6 Hz, 1H); 7.59 (t, *J* = 7.3 Hz, 1H); 7.84 (d, *J* = 7.8 Hz, 1H); 8.24 (d, *J* = 7.7 Hz, 1H); 8.51 (t, *J* = 5.9 Hz, 1H); 8.78 (s, 1H); 11.58 (s, 1H); 13.26 (br. s, 1H).

^13^C-NMR (125 MHz, DMSO-d_6_, ppm) δ: 25.84; 25.91; 28.17; 28.66; 43.37; 50.58; 127.05; 129.25; 130.04; 130.75; 131.60; 134.32; 141.00; 168.17; 177.04.

IR (ATR, cm^−1^) $\bar{\nu }$: 3359 (ν_NH_); 3174 (ν_NH_); 2095 (ν_-N=N=N_); 1538 (ν_C=N_)

#### 2.3.2. Electrode Preparation

The GC electrodes coated with electrochemically reduced graphene oxide (further denoted GC-ERGO) were prepared following the procedure described in ref. [30].

The modification of GC and GC-ERGO electrodes was performed using the protocol described in our previous publication [29]. Briefly, electrodes were first grafted with phenylethynyl groups by potential cycling in a 1 mM solution of 4-ethynylphenyldiazonium tetrafluoroborate in MeCN (3 cycles between 0.3 and −0.25 V, scan rate 0.1 V s^−1^). Next, ligand **L** was immobilized by immersing the electrodes for 24 h in a mixture of DMF:TEA (2.5:1 *v*/*v*) containing 0.5 mM **L** and 2 mM CuBr(PPh_3_)_3_. The modified electrodes (further denoted GC|click|**L** and GC-ERGO|click|**L**) were thoroughly washed with DMF and water, dried, and then kept in sealed tubes until further use. 

GC disk electrodes with a diameter of 6 mm were used for SEM, XPS, and Raman spectroscopy investigations, and their modification followed the same procedure described above.

Voltammetric determinations with the modified electrodes were performed through a chemical accumulation step at open circuit followed by anodic stripping using differential pulse voltammetry (DPV) [29,31,32]. The DPV curves were recorded at 20 mV s^−1^ with an amplitude of 25 mV and 0.5 s pulse period. 

The quantitation of Hg(II) in water samples was performed in an accredited laboratory, according to standardized methods: VG-AAS (calibration range 1–10 µg/L, limit of quantification 0.5 µg/L, precision 2.2%, uncertainty 11%) [33] and CV-AFS (calibration range 0.02–0.1 µg/L, limit of quantification 0.01 µg/L, precision 3.1%, uncertainty 10%) [34].

## 3. Results and Discussion

### 3.1. Preparation of Modified Electrodes

**Scheme 1:**
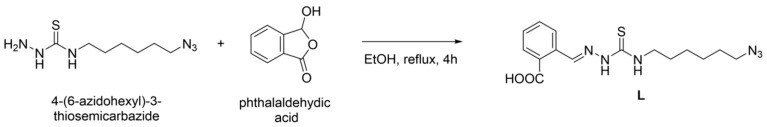
Synthesis of thiosemicarbazone ligand **L**.

A novel thiosemicarbazone ligand (further denoted **L**) was synthesized by reacting 4-(6-azidohexyl)-3-thiosemicarbazide [29] with phthalaldehydic acid (Scheme 1). 

**Figure 1:**
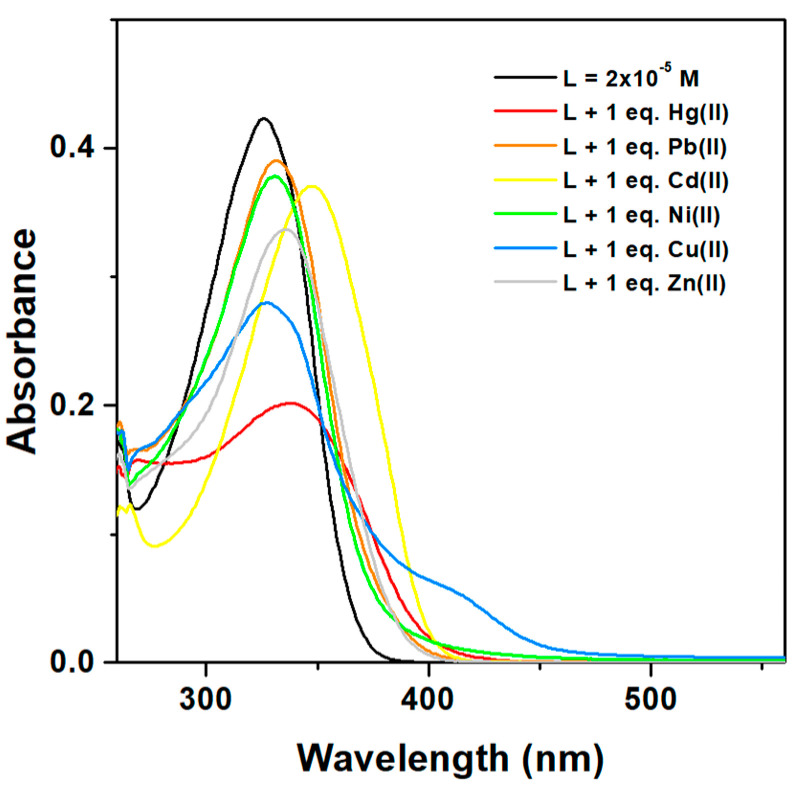
UV-VIS spectra of ligand **L** before and after the addition of 1 equivalent of heavy metal ions.

The UV-Vis spectra of ligand **L** recorded in the presence of 1 equivalent of different heavy metal ions (Figure 1) show a bathochromic and hypochromic shift of the absorption band at 326 nm. The most significant spectral changes occur in the presence of Hg(II) and Cu(II), which suggests that the ligand has an increased affinity for these ions. This behavior resembles that of analogous thiosemicarbazone ligands synthesized in our laboratory [29]. However, during a preliminary assessment of several azido-functionalized thiosemicarbazones, we observed a decreased sensibility of GC electrodes modified with ligand **L** for the voltammetric detection of Hg(II) ions. This fact prompted us to investigate an alternative electrode substrate, i.e., ERGO, in order to improve the analytical performance of the modified electrodes. 

We found a single reference describing the analytical applications of a thiosemicarbazone derived from phthalaldehydic acid [35], and apparently that ligand showed a lower affinity for Hg(II) among several metal ions, in contrast to thiosemicarbazones described in our previous study [29]. This is not surprising, since the relative stability of metal complexes depends on particular structural features of the ligand molecule. For example, it has been shown that thiosemicarbazones can act as either bidentate ligands, binding metal ions through the sulfur and the hydrazine nitrogen atoms, or tridentate species if additional coordinating groups are present (e.g., -OH groups, pyridine N) [36]. Moreover, the formation of complexes with phenyl-substituted thiosemicarbazone ligands can be influenced by noncoordinating functional groups, as the thiol-thione equilibrium is altered by electronic effects [37]. 

Electrochemically reduced graphene oxide (ERGO) was deposited onto glassy carbon (GC) substrates through the potentiodynamic reduction (10 cycles between 0.5 and −1.5 V, 20 mV s^−1^) of a deaerated aqueous dispersion of graphene oxide (1 mg mL^−1^ in pH 9.2, 0.067 M phosphate buffer) [30].

**Scheme 2:**
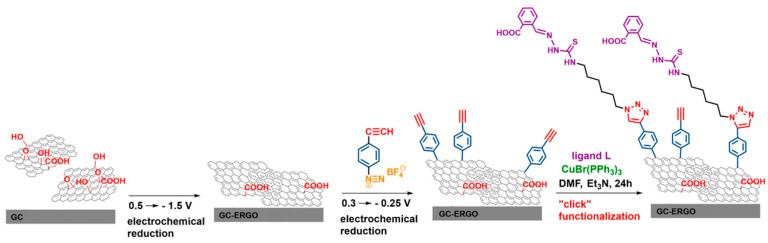
Preparation of GC-ERGO|click|**L** modified electrodes.

Next, the surface modification of GC and GC-ERGO electrodes was performed using the two-step protocol described in our previous paper [29] (Scheme 2). The first step consists in grafting phenylethynyl groups on the electrode surface, by cycling the potential in a solution containing the corresponding diazonium salt. The second step consists of attaching ligand **L**, which contains an azido group, to the alkyne-functionalized electrode through a Cu(I)-catalyzed azide-alkyne cycloaddition reaction.

**Figure 2:**
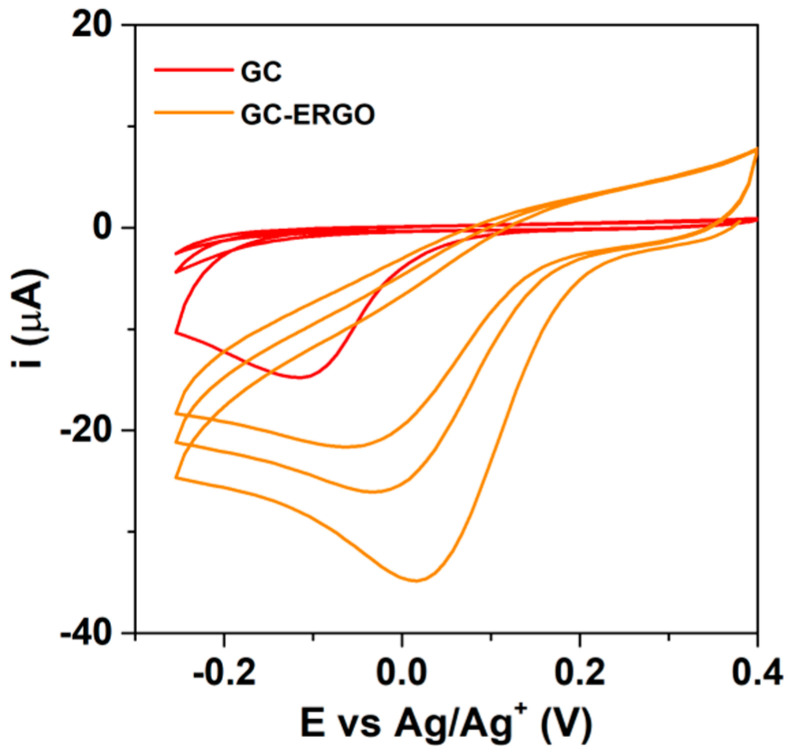
Cyclic voltammograms recorded in a 1 mM solution of 4-ethynylphenyldiazonium tetrafluoroborate (100 mV s^−1^) at GC and GC-ERGO electrodes.

The cyclic voltammograms corresponding to the reduction of 4-ethynylphenyldiazonium tetrafluoroborate (1 mM in MeCN with 0.1 M TBABF_4_) on GC and GC-ERGO electrodes are shown in Figure 2. In both cases, the first scan displays a broad and irreversible cathodic peak, but on GC-ERGO the peak current is larger (32 μA vs. 14 μA) and the peak potential is shifted anodically (0.02 vs. −0.11 V). 

For the GC electrode, this peak disappears in the second scan since the reduction of aryldiazonium salts is self-inhibited by the grafted layers [38]. However, in the case of GC-ERGO, during the subsequent scans there is only a ~25% decrease in the peak current after each cycle, accompanied by a cathodic shift of the peak potential. 

We believe the contrasting behavior of GC and GC-ERGO can be explained by considering the nature of the two electrodes, which consist of basal plane and edge plane surfaces [39]. Although reports concerning the electrochemical activity of the basal plane vs. edge plane of graphite-like materials are somewhat contradictory, recent results obtained using localized electrochemical measurements prove unequivocally that the edges and defects are more active than the basal plane, even though the latter has a non-negligible electrochemical activity [40]. This difference in reactivity was also observed in the case of the electrochemical reduction of aryldiazonium salts [38], and it was demonstrated through spatially resolved Raman spectroscopy and scanning tunneling microscopy (STM) that diazonium grafting of highly oriented pyrolitic graphite (HOPG) results in the deposition of more material at the edge planes than at the basal plane [41,42]. Moreover, in the case of graphene functionalization with diazonium salts, experimental evidence obtained using spatially and temporally resolved Raman mapping showed that nucleation sites are located on edges, and grafted regions spread from the edges toward the interior of the flakes [43,44].

It is well known that aryl radicals resulting from the electrochemical reduction of diazonium salts can react with the electrode surface or with other aryl groups already bound to the surface, leading to poly(aryl) films that grow in both two- and three-dimensions [45]. Because GC has an increased density of edge-plane sites [46], a more uniform grafting across the electrode surface leads to increased barrier properties of the aryl layers, consistent with the rapid drop in current observed in the cyclic voltammograms. Conversely, on a GC-ERGO surface dominated by lower activity basal planes, aryl radicals will react preferentially with other aryl groups and not with the surface, leading to a three-dimensional irregular growth of the layers. Presumably, such layers contain more defects (i.e., discontinuities, pores) and are less blocking, which explains the increased peak current observed in all the voltammetric cycles. There is experimental evidence that the blocking behavior of aryl multilayers does not depend on layer thickness, presumably due to their nonuniform topography [47]. At the same time, the increased amount of charge consumed during the reduction of diazonium salt at the GC-ERGO electrode suggests that in this case a higher amount of material is deposited onto the electrode surface. 

### 3.2. Characterization of Modified Electrodes

**Figure 3:**
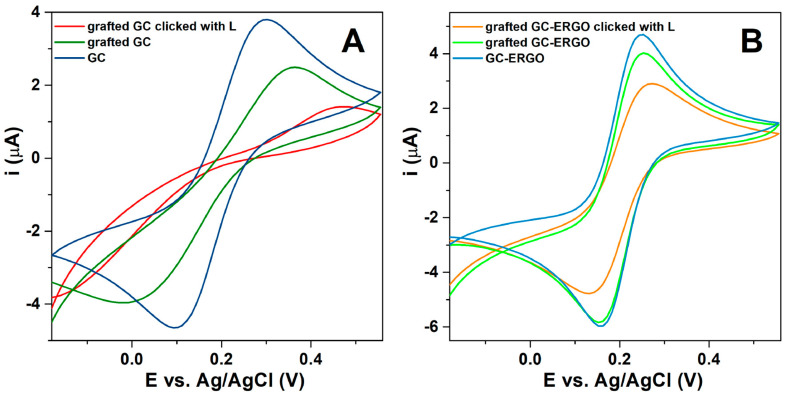
Cyclic voltammograms recorded in 1 mM Fe(CN)_6_^3−^, 0.1 M KCl solution (20 mV s^−1^), at (**A**) GC and (**B**) GC-ERGO electrodes, after different modification steps.

The electrode functionalization steps were monitored through cyclic voltammetry using the ferricyanide and ferrocenemethanol redox probes [48]. Since the voltammograms (Figure 3 and Figure S3 from Supporting Information) display a quasireversible behavior of the redox couples, the peak current $i_{p}$ is given by a modified version of the Randles–Ševčík equation [49]:

(1) $$i_{p}=\left(2.69\times 10^{5}\right)n^{3/2}AD^{1/2}Cv^{1/2}K\left(\Lambda ,\alpha \right)$$

where n is the number of transferred electrons, A the electrode area (cm^2^), D the diffusion coefficient of the redox probe (cm^2^ s^−1^), C the bulk concentration of the redox probe (mol cm^−3^), v the scan rate (V s^−1^) and K(Λ,α) is a function that depends on the rate parameter Λ and the charge transfer coefficient α [50]. The rate parameter Λ is defined as:

(2) $$\Lambda =\pi^{1/2}\Psi =k^{{}^{\circ}}{\left(D_{O}^{1-\alpha }D_{R}^{\alpha }nfv\right)}^{-1/2}$$

where ψ is a dimensionless charge transfer parameter [51], $k^{{}^{\circ}}$ is the standard heterogeneous rate constant (cm s^−1^), $D_{O}$ the diffusion coefficient of the oxidized species, $D_{R}$ the diffusion coefficient of the reduced species, and f=F/RT with F, R and T having their usual meaning. 

The parameters ψ and K(Λ,α) were estimated using the equations given in ref. [52] and Figure 4 from ref. [50], respectively. The values of the diffusion and transfer coefficients were taken from the literature: for Fe(CN)_6_^3−^, α=0.5, $D_{O}=7.6\times 10^{-6}$ cm^2^ s^−1^, $D_{R}=6.5\times 10^{-6}$ and for FcMeOH α=0.5 and $D_{O}=D_{R}=7.8\times 10^{-6}$ cm^2^ s^−1^ [53,54].

There are no major differences in the electrochemical response of the FcMeOH^0/+^ on the unmodified GC and GC-ERGO electrodes (Figure S3 from Supporting Information), so this redox probe was employed mainly for determining electroactive surface areas, which are 6.9 × 10^−2^ cm^2^ for GC and 9.9 × 10^−2^ cm^2^ for the ERGO electrode substrate, respectively. It should be noted that experimental evidence presented in the following sections suggests that the 43% increase in surface area determined for GC-ERGO is not solely responsible for the contrasting behavior of the two modified electrodes.

Because the Fe(CN)_6_^3−^ electron transfer kinetics is very sensitive to surface chemistry [55], it is more suitable to monitor electrode modification. As expected, the heterogeneous electron transfer rate constant ($k^{{}^{\circ}}$, Table 1) decreases by a factor of ~3 following the GC electrode modification with phenylethynyl groups. Interestingly, $k^{{}^{\circ}}$ decreases almost 15 times after performing the click functionalization step. 

On the contrary, when using GC-ERGO electrodes as substrates, the Fe(CN)_6_^3−^ electron transfer kinetics does not show significant changes (Figure 3B), as $k^{{}^{\circ}}$ decreases only ~1.5 times after grafting and again ~2.5 times after performing the click reaction. This indicates that a modified GC-ERGO surface is significantly less blocked ($k^{{}^{\circ}}$ is two orders of magnitude higher), which is entirely consistent with the behavior during grafting with diazonium salt.

**Figure 4:**
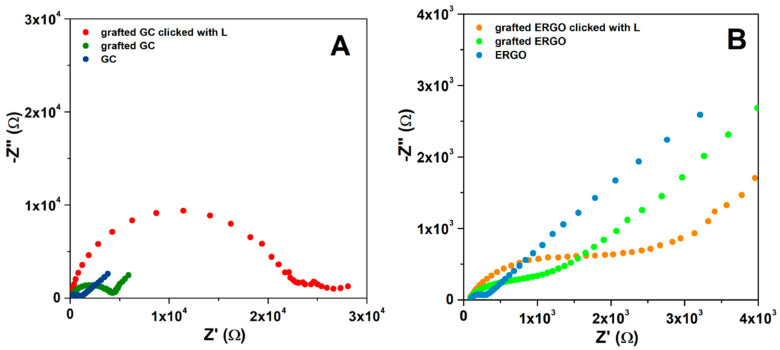
Electrochemical impedance spectra (Nyquist plots) recorded in 5 mM Fe(CN)_6_^3−/4−^, 0.1 M KCl solution, at (**A**) GC and (**B**) GC-ERGO electrodes, at different modification steps.

Electrochemical impedance spectroscopy (EIS) in the presence of Fe(CN)_6_^3−/4−^ as redox probe was further used to evaluate the surface modification of GC and GC-ERGO electrodes. The corresponding Nyquist plots are shown in Figure 4, and charge-transfer resistance ($R_{\mathrm{CT}}$) values were obtained by fitting the data with a Randles equivalent circuit [56]. The charge-transfer resistance increases from ~0.9 kΩ to ~3.8 kΩ after grafting the GC electrode with phenylethynyl groups. Again, a significant blocking can be observed following the click functionalization step, as $R_{\mathrm{CT}}$ increases to ~22.5 kΩ. In the case of GC-ERGO electrode, $R_{\mathrm{CT}}$ increases after grafting from 0.18 kΩ to 1.18 kΩ, and after the second step to 2.24 kΩ. It should be noted that the $R_{\mathrm{CT}}$ value for the GC-ERGO modified electrode is lower by one order of magnitude than the GC modified electrode, in agreement with the CV results. 

**Figure 5:**
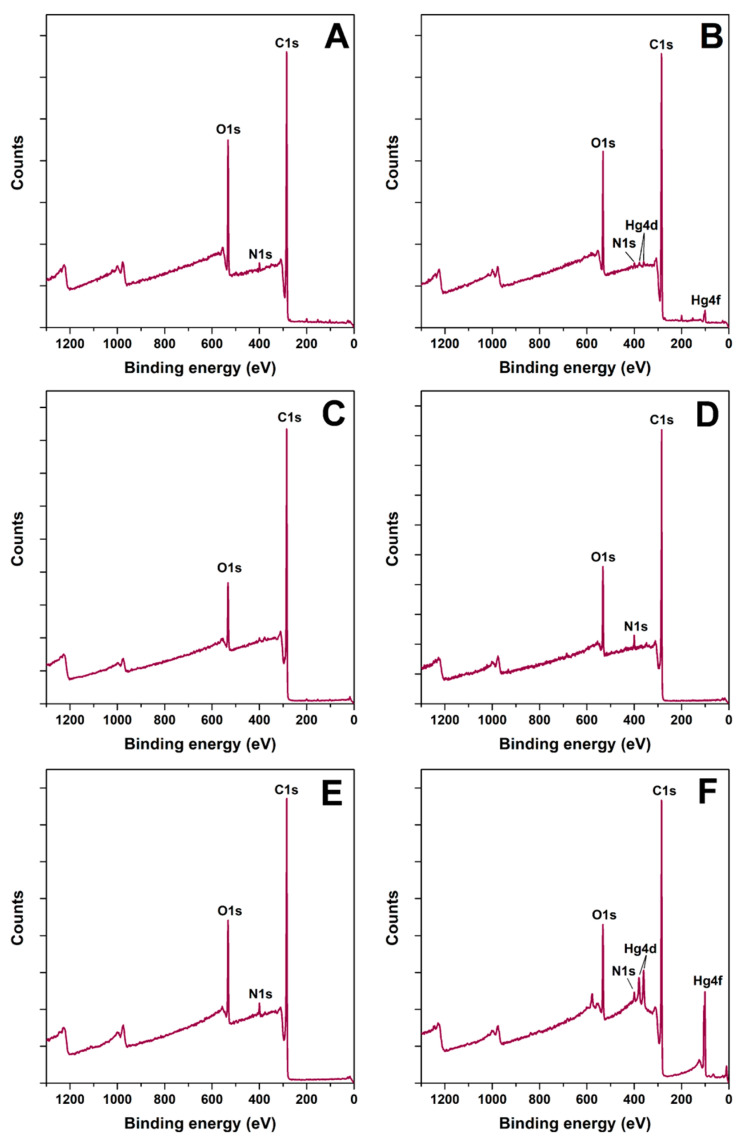
XPS survey spectra of GC and GC-ERGO electrodes after various modification steps: (**A**) GC|click|**L**; (**B**) GC|click|**L** treated in a Hg(II) solution; (**C**) GC-ERGO; (**D**) GC-ERGO grafted with phenylethynyl groups; (**E**) GC-ERGO|click|**L**; (**F**) GC-ERGO|click|**L** treated in a Hg(II) solution.

Next, the surface of the modified GC and GC-ERGO electrodes was investigated using XPS, and the corresponding survey spectra are shown in Figure 5. The C/N atomic ratios presented in Table 2 seem to indicate a higher degree of functionalization for GC-ERGO|click|**L** (C/N = 37.2) than GC|click|**L** (C/N = 64.2), which correlates with the increased amount of charge consumed during grafting with diazonium salt. Moreover, when the two electrodes were immersed in a solution containing 10^−6^ M Hg(II) for 1 h, washed and then analyzed using XPS, the C/Hg ratio is ~2 times lower for GC-ERGO|click|**L** than GC|click|**L** although the N/Hg ratio is the same for both electrodes. Undoubtedly a higher degree of surface modification with phenylethynyl groups led to an increased concentration of surface-bound ligand **L**.

The electrode surface modification was also assessed using Raman spectroscopy. The Raman spectra (Figure S4 from Supporting Information) display the G band (~1580 cm^−1^) characteristic for the sp^2^-hybridized carbon lattice and the defect-related D band (~1360 cm^−1^), the intensity ratio I_D_/I_G_ being related to the number of sp^3^ defect sites [57]. After functionalization both the GC and GC-ERGO electrodes show an increase in the I_D_/I_G_ ratio, from 1.31 to 1.41 in the former case and from 1.02 to 1.26 in the latter case, which confirms the covalent attachment of aryl radicals generated through the electrochemical reduction of diazonium salt. Because the differences between the I_D_/I_G_ ratios before and after modification are quite similar, and taking into account the greater amount of grafting in the case of GC-ERGO|click|**L**, supports the idea of a three-dimensional layer growth in the case of graphene.

The surface morphology of unmodified and modified GC-ERGO electrodes was further examined using SEM (Figure S5A,B from Supporting Information). The micrographs reveal an incomplete coverage of the GC substrate with agglomerates consisting of stacked ERGO sheets, in line with previous observations [58]. Moreover, there seems to be no apparent difference between the electrode surface morphology before and after modification. Interestingly, the micrograph of an GC-ERGO|click|**L** electrode treated in a 10^−6^ M Hg(II) solution and imaged using backscattered electrons (Figure S5C) suggests that ERGO agglomerates, which appear lighter, contain an increased amount of mercury. 

### 3.3. Voltammetric Detection of Hg(II)

**Figure 6:**
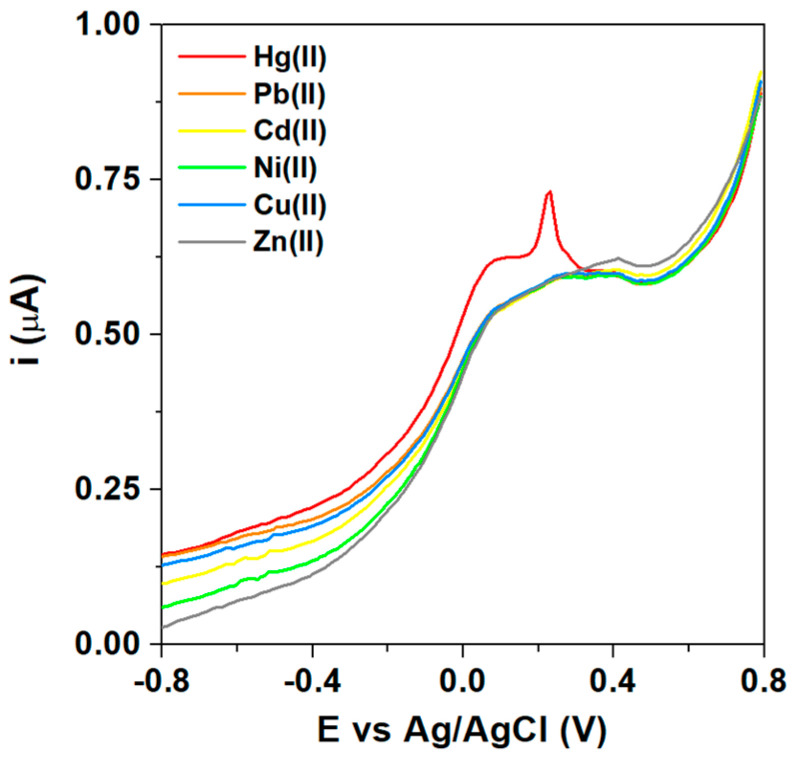
Differential pulse voltammetry (DPV) curves recorded on a GC|click|**L** modified electrode after 10 min accumulation in acetate buffer (pH 4.5) containing 5 × 10^−8^ M of various heavy metal ions.

Furthermore, the complexation of several heavy metal ions in heterogenous phase was investigated using GC|click|**L** modified electrodes. Figure 6 shows the response of the modified electrode after 10 min accumulation at open circuit in acetate buffer (pH 4.5). The GC|click|**L** electrode shows a DPV response only for mercury ions, which appear as a stripping peak at ~0.24 V. Considering the selectivity for Hg(II), which seems to be characteristic for electrodes modified with thiosemicarbazone ligands when the complexation is performed at open circuit [29], the performances of GC|click|**L** were further evaluated only for this ion. 

**Figure 7:**
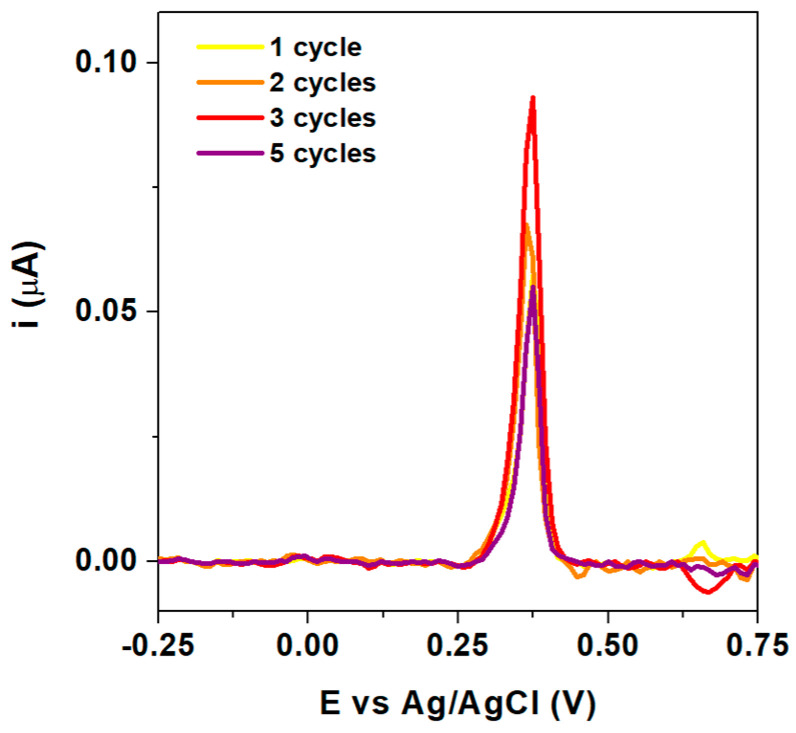
DPV curves (background corrected) recorded in a solution containing 5 × 10^−8^ M Hg(II) on GC|click|**L** modified electrodes prepared using different number of scans during grafting with diazonium salt.

Next, in order to investigate the effect of electrode preparation conditions on the Hg(II) response, DPV curves were recorded in a 5 × 10^−8^ Hg(II) solution on several GC|click|**L** modified electrodes prepared using a different number of potential cycles during grafting with diazonium salt (Figure 7). After an initial increase up to three potential cycles, the stripping current starts to drop. This observation implies that two opposite effects influence the electrode response: the amount of surface-bound ligand **L** increases with the number of potential cycles employed for grafting, leading to an enhanced stripping current, but at the same time the layers become more blocking and the stripping current decreases when the grafted layer becomes too thick. It should be noted at this point that, in order to allow for a meaningful comparison, identical functionalization conditions were employed for both GC and GC-ERGO substrates, although in the latter case the improved electron transfer properties of the grafted layer suggest that the analytical performance can be further enhanced by increasing the amount of surface-bound ligand.

The analytical response GC|click|**L** modified electrodes towards Hg(II) was further optimized considering several analytical parameters. Because the pH has a major influence on the formation of complexes with thiosemicarbazone ligands [36] and at the same time determines the mercury speciation [59], we investigated the GC|click|**L** electrode response in the pH range 3 to 6 (Figure S6A from Supporting Information). Since the best response was attained at pH 4, this value was maintained for the analytical determinations. Above this pH value, the Hg(II) peak current decreases slightly, probably as a consequence of hydrolysis reactions which lead to basic salts [60]. This is in agreement with previous observations [29,61]. Furthermore, a steady increase of the stripping current was observed for accumulation times up to 10 min (Figure S6B from Supporting Information), suggesting that at this point most surface sites become occupied by Hg(II) ions. To achieve the best sensitivity, an accumulation time of 15 min was chosen for analytical determinations. Finally, we investigated the influence of the reduction potential and reduction time on the stripping current, and we found that a potential of −0.9 V and a time of 180 s allowed the complete reduction of accumulated mercury ions (Figure S6C,D from Supporting Information).

**Figure 8:**
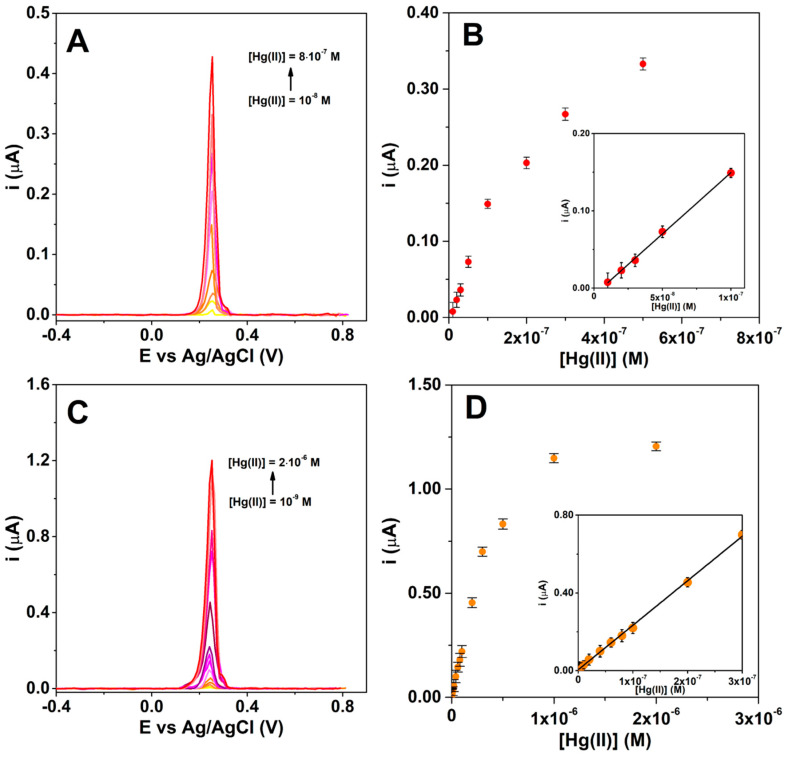
Typical DPV curves (**A**,**C**) and calibration plots (**B**,**D**) for Hg(II) analysis with GC|click|**L** (**A**,**B**) and GC-ERGO|click|**L** (**C**,**D**) modified electrodes. All DPV curves are background corrected.

The analytical performance of the modified electrodes was further assessed using the optimized DPV method (Figure 8 and Table 3). The GC|click|**L** electrodes exhibit a narrow linear dependence between the stripping peak current and Hg(II) concentration (Figure 8B) and a relatively good limit of detection (LOD, estimated as 3-times signal-to-noise ratio). However, when the same analyses were performed with GC-ERGO|click|**L** electrodes (Figure 8C), the analytical performance was substantially improved (Table 3). These electrodes showed a wider linear range, a LOD which is ten times lower and a two-fold increase in sensitivity (Figure 8D).

We believe the improved linearity at the lower range of Hg(II) concentrations and lower LOD obtained with GC-ERGO electrodes are consistent with an increased amount of surface-bound ligand, which facilitates Hg(II) adsorption, leading in turn to enhanced stripping currents. This would be in agreement with the CV, XPS, SEM, and Raman spectroscopy results discussed in the previous sections. Moreover, this might also explain the improved linearity at higher analyte concentrations, as it is well known that deviations from linearity appear when surface sites become saturated [62].

### 3.4. Reproducibility, Interference, and Real Sample Analysis

The reproducibility concerns both electrode preparation and the possibility of reusing the same electrode for repeated Hg(II) determinations. Consequently, five GC and five GC-ERGO electrodes were functionalized with **L** and employed for the analysis of a solution containing 8 × 10^−8^ M Hg(II). Relative standard deviations (RSD) of 3.56% and 4.26% were obtained for the GC|click|**L** and GC-ERGO|click|**L** electrodes, respectively, which indicate a good reproducibility of the electrode preparation procedure. 

Undoubtedly, the possibility of using the same electrode for multiple determinations is also an important factor when considering a new electroanalytical method. As previously reported for electrodes modified with thiosemicarbazone ligands [29], an effective regeneration of GC|click|**L** and GC-ERGO|click|**L** electrodes was achieved by competitive chemical decomplexation in a solution of sodium diethyldithiocarbamate (Figure S7 from Supporting Information). For the GC|click|**L** electrode, RSDs of 4.13% and 12.9% were obtained after 5 and 10 consecutive Hg(II) determinations. Rather similar values were observed for the GC-ERGO|click|**L** electrode, where RSDs of 4.92% and 13.6% were attained after 5 and 10 measurements, respectively. This indicates that both modified electrodes can be used for up to 5 Hg(II) determinations, after which the regeneration treatment starts to affect the electrode surface.

**Figure 9:**
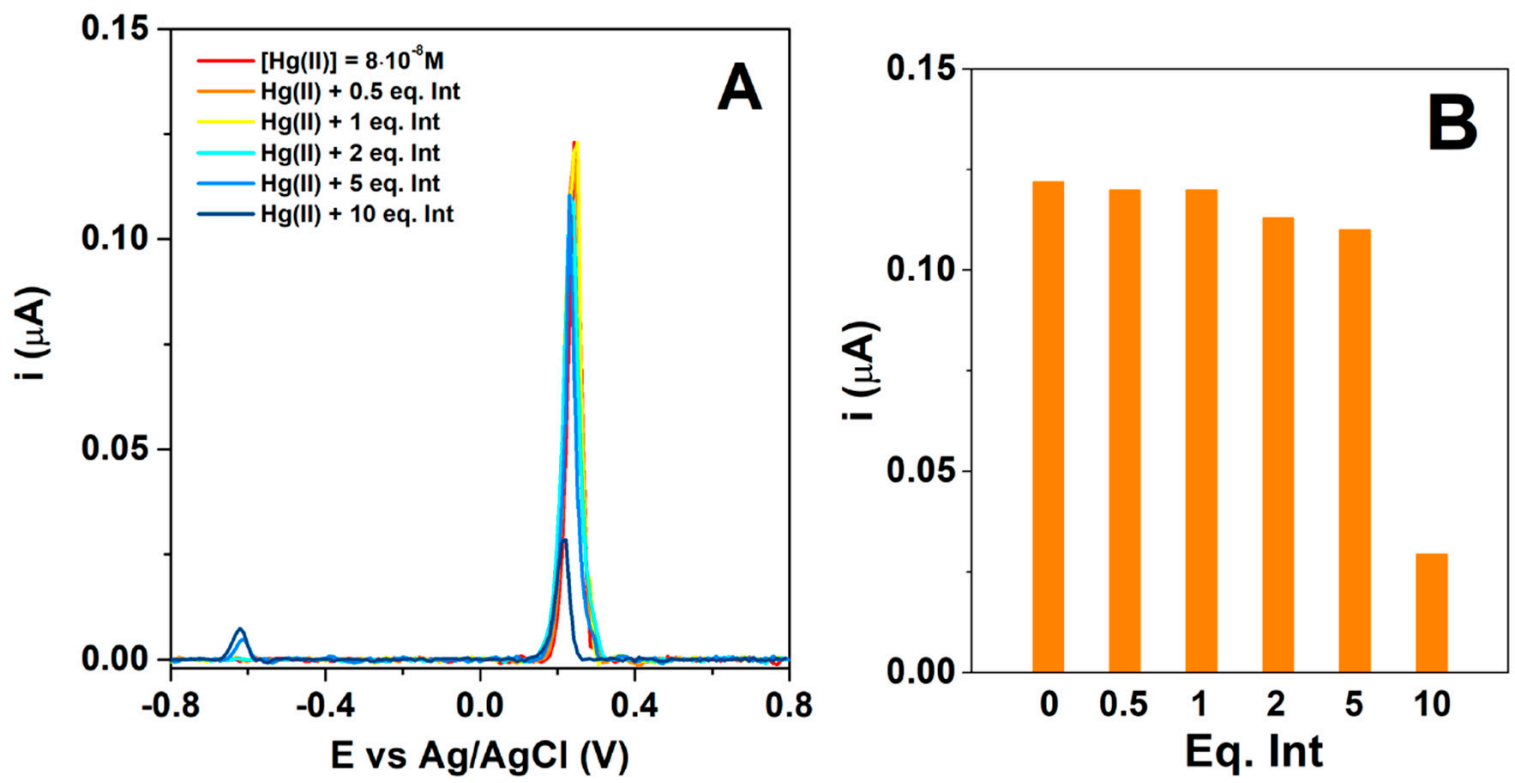
DPV curves (background corrected) recorded on a GC|click|**L** modified electrode (**A**) and Hg(II) stripping current intensity (**B**) in the presence of a mixture of interfering ions.

Next, the interfering effect of heavy metal ions usually found in water (a mixture of Zn^2+^, Cd^2+^, Pb^2+^, Ni^2+^, and Cu^2+^, further denoted *Int*) on the analysis of solutions containing 8 × 10^−8^ M Hg^2+^ was investigated. As can be seen in Figure 9, the Hg(II) stripping current deviates less than ±5% up to an excess of five equivalents of *Int*. At higher concentrations of *Int*, the stripping current decreases and a new peak appears at −0.6 V, which we attribute to the redissolution of Pb(II) ions retained on the electrode surface. This indicates that a part of the surface sites become occupied by Pb(II) ions, leading to a decrease in the amount of Hg(II) ions accumulated on the electrode surface.

Lastly, the detection of mercury ions from tap water samples was performed using the modified electrodes, and results were compared with values obtained using standard AAS and AFS methods (Table 4). The good correlation between these methods, with a recovery between 97% and 103%, proves that both GC|click|**L** and GC-ERGO|click|**L** electrodes can be employed successfully for the anodic stripping voltammetric determination of mercury ions in real water samples. 

The uncertainty budget for the voltammetric determinations was evaluated taking into account both electrode preparation and the voltammetric determination procedures. The obtained uncertainty values are 18.6% and 21.0% for the GC|click|**L** and GC-ERGO|click|**L** electrodes, respectively. Furthermore, the RSD of the voltammetric determinations falls below the limits recommended in the literature, i.e., a maximum RSD of 45.3% for 1 µg/L and 32% for 10 µg/L [63]. Finally, the recovery percentages for all the analyzed samples fall in the recommended range, which is between 40% and 120% for 1 µg/L, and between 60% and 115% for 10 µg/L [63].

## 4. Conclusions

Glassy carbon (GC) and electrochemically reduced graphene oxide (ERGO) electrodes modified with a new thiosemicarbazone ligand were prepared using a two-step functionalization protocol which involves surface grafting with phenylethynyl groups via the corresponding diazonium salt and the subsequent ligand immobilization through an azyde-alkyne cycloaddition catalyzed by Cu(I). The extent of surface modification was assessed through various techniques, and we demonstrate that in the case of ERGO, the diminished self-inhibition of diazonium reduction allows a higher degree of surface functionalization and leads to improved electron transfer properties of the grafted layers.

Using the accumulation at open circuit followed by anodic stripping voltammetry for the determination of Hg(II) ions, we demonstrate that ERGO outperforms GC as a substrate for obtaining ligand-modified electrodes, at least when the modification strategy is based on aryldiazonium chemistry. In the case of ERGO, the increased number of surface binding sites extends the linearity domain in both lower and upper analyte concentration ranges, and, at the same time, the reduced blocking effect of the grafted layers is probably responsible for the improved sensitivity of these electrodes.

## Figures and Tables

**Table 1:** Electrochemical parameters determined from cyclic voltammograms recorded in 0.5 mM FcMeOH, 0.1 M KCl and 1 mM Fe(CN)_6_^3−^, 0.1 M KCl solutions, at GC and GC-ERGO electrodes, after various modification steps.

Electrode	Redox Probe	$E_{p,a}$ (V)	$E_{p,c}$ (V)	$\Delta E_{p}$ (V)	$i_{p,a}$ (A)	$i_{p,c}$ (A)	ψ	Κ(Λ,α)	$k^{{}^{\circ}}$ (cm s−1)
GC	FcMeOH	0.20	0.12	0.08	3.4 × 10^−6^	3.4 × 10^−6^	1.3	0.96	5.6 × 10^−3^
GC-ERGO		0.21	0.14	0.07	5.2 × 10^−6^	5.2 × 10^−6^	2.5	0.98	1.1 × 10^−2^
GC	Fe(CN)6 ^3−^	0.30	0.10	0.20	5.4 × 10^−6^	5.5 × 10^−6^	0.087	-	3.6 × 10^−4^
GC grafted ^a)^		0.36	0.02	0.34	3.7 × 10^−6^	4.1 × 10^−6^	0.031	-	1.3 × 10^−4^
GC|click|**L**		0.46	−0.17	0.63	-	-	0.002	-	8.3 × 10^−6^
GC-ERGO		0.24	0.15	0.09	6.8 × 10^−6^	6.8 × 10^−6^	0.83	-	3.4 × 10^−3^
GC-ERGO grafted ^a)^		0.25	0.15	0.10	6.3 × 10^−6^	6.3 × 10^−6^	0.59	-	2.4 × 10^−3^
GC-ERGO|click|**L**		0.27	0.13	0.14	5.1 × 10^−6^	5.2 × 10^−6^	0.24	-	9.9 × 10^−4^

^a)^ electrode grafted with phenylethynyl groups.

**Table 2:** Atomic composition of the electrode surface after various modification steps.

Electrode	Atomic Concentration (%)				Atomic Ratios			
	C 1s	O 1s	N 1s	Hg 4f	C/O	C/N	C/Hg	N/Hg
GC|click|**L**	83.0	15.9	1.1	-	5.6	64.2	-	-
GC|click|**L** + Hg(II) ^a)^	82.9	15.3	1.1	0.7	5.6	60.8	106.9	1.2
GC-ERGO	90.6	8.2	1.2	-	10.4	68.3	-	-
GC-ERGO grafted ^b)^	86.9	11.0	2.1	-	7.3	34.0	-	-
GC-ERGO|click|**L**	85.2	12.3	2.5	-	6.8	37.2	-	-
GC-ERGO|click|**L** + Hg(II) ^a)^	85.1	11.6	2.0	1.3	7.1	34.5	42.6	1.2

^a)^ electrode treated in a solution containing Hg(II) ions and then washed; ^b)^ GC-ERGO electrode grafted with phenylethynyl groups.

**Table 3:** Analytical performance of GC|click|**L** and GC-ERGO|click|**L** modified electrodes for Hg(II) sensing.

Electrode	Linearity Domain (M)	Linear Regression Equation	Limit of Detection (M)
GC|click|**L**	10^−8^ ÷ 10^−7^	i (A) = 8 × 10^−9^ + 1.58C (M); R^2^ = 0.998	7 × 10^−9^
GC-ERGO|click|**L**	10^−9^ ÷ 3 × 10^−7^	i (A) = 4.5 × 10^−9^ + 2.3C (M); R^2^ = 0.9996	8 × 10^−10^

**Table 4:** Determination of Hg(II) in tap water using GC|click|**L** and GC-ERGO|click|**L** modified electrodes. For comparison, Hg(II) concentrations were also determined using standard AAS and AFS methods.

Sample	Added Hg(II) (M)	Method			
		GC|Click|L	GC-ERGO|Click|L	VG-AAS	CV-AFS
Tap water	-	ND ^a^	ND	ND	ND
Spiked tap water I	2.5 × 10^−8^	2.47 (±0.46) × 10^−8^	2.48 (±0.52) × 10^−8^	2.61 (±0.31) × 10^−8^	2.54 (±0.25) × 10^−8^
Spiked tap water II	1 × 10^−7^	0.97 (±0.18) × 10^−^^7^	0.95 (±0.20) × 10^−^^7^	0.93 (±0.11) × 10^−^^7^	1.02 (±0.10) × 10^−^^7^

^a^ Not detected.

## Supplementary Materials

The following are available online at https://www.mdpi.com/1424-8220/20/23/6799/s1, Figure S1: ^1^H-NMR (500 MHz) and ^13^C-NMR (125 MHz) spectra of thiosemicarbazone ligand **L** (DMSO-d_6_), Figure S2: Infrared spectrum of thiosemicarbazone ligand **L**, Figure S3: Cyclic voltammograms recorded in 1 mM ferrocenemethanol, 0.1 M KCl solution (20 mV s^−1^), on (A) GC and (B) GC-ERGO electrodes, Figure S4: Raman spectra of (A) GC, (B) GC|click|**L**, (C) GC-ERGO and (D) GC-ERGO|click|**L** electrodes, Figure S5: SEM micrographs of the electrode surfaces for A) GC-ERGO, B) GC-ERGO|click|**L**, C) GC-ERGO|click|**L** treated in a Hg(II) solution (backscattered electron detector), Figure S6: The effect of (A) pH of the accumulation solution, (B) accumulation time at open circuit, (C) deposition potential and (D) time for the reduction of accumulated ions, on the Hg(II) stripping current recorded with a GC|click|**L** modified electrode, Figure S7: Hg(II) DPV curves recorded on GC|click|**L** electrodes before and after decomplexation in 1 mM sodium diethyldithiocarbamate solution.
