# Supplementary material for: Improving the Voltammetric Determination of Hg(II): A Comparison Between Ligand-Modified Glassy Carbon and Electrochemically Reduced Graphene Oxide Electrodes
Source: Sensors (Basel). 2020 Nov 28;20(23):6799. doi: 10.3390/s20236799 (PMC7729478; doi:10.3390/s20236799)
Supplement: Supplementary file 1 [file sensors-20-06799-s001.pdf]

SUPPORTING INFORMATION

Improving the voltammetric determination of Hg(II): A comparison between ligand-modified glassy carbon and electrochemically reduced graphene oxide electrodes

Matei D. Raicopol[1], Andreea M. Pandele[1], Constanţa Dascălu[2], Eugeniu Vasile[1], Anamaria Hanganu[3], Gabriela-Geanina Vasile[4], Ioana Georgiana Bugean[1], Cristian Pirvu[1], Gabriela Stanciu[5*], George-Octavian Buica[1*]

[1] University Politehnica of Bucharest, Faculty of Applied Chemistry and Materials Science, 1-7 Gheorghe Polizu St., 011061, Bucharest, Romania

[2] University Politehnica of Bucharest, Faculty of Applied Sciences, 313 Splaiul Independenţei, 060042, Bucharest, Romania

[3] University of Bucharest, Department of Organic Chemistry, Biochemistry and Catalysis, 90-92 Sos. Panduri, 050657, Bucharest, Romania

[4] National Research and Development Institute for Industrial Ecology ECOIND Bucharest, 71-73 Drumul Podul Dambovitei Street, Bucharest, 060652, Romania

[5] Ovidius University, Department of Chemistry and Chemical Engineering, 124 Mamaia Blvd, Constanta, 900527, Romania

* Correspondence: buica_george@yahoo.com (G.-O.B.); gstanciu@univ-ovidius.ro (G.S)

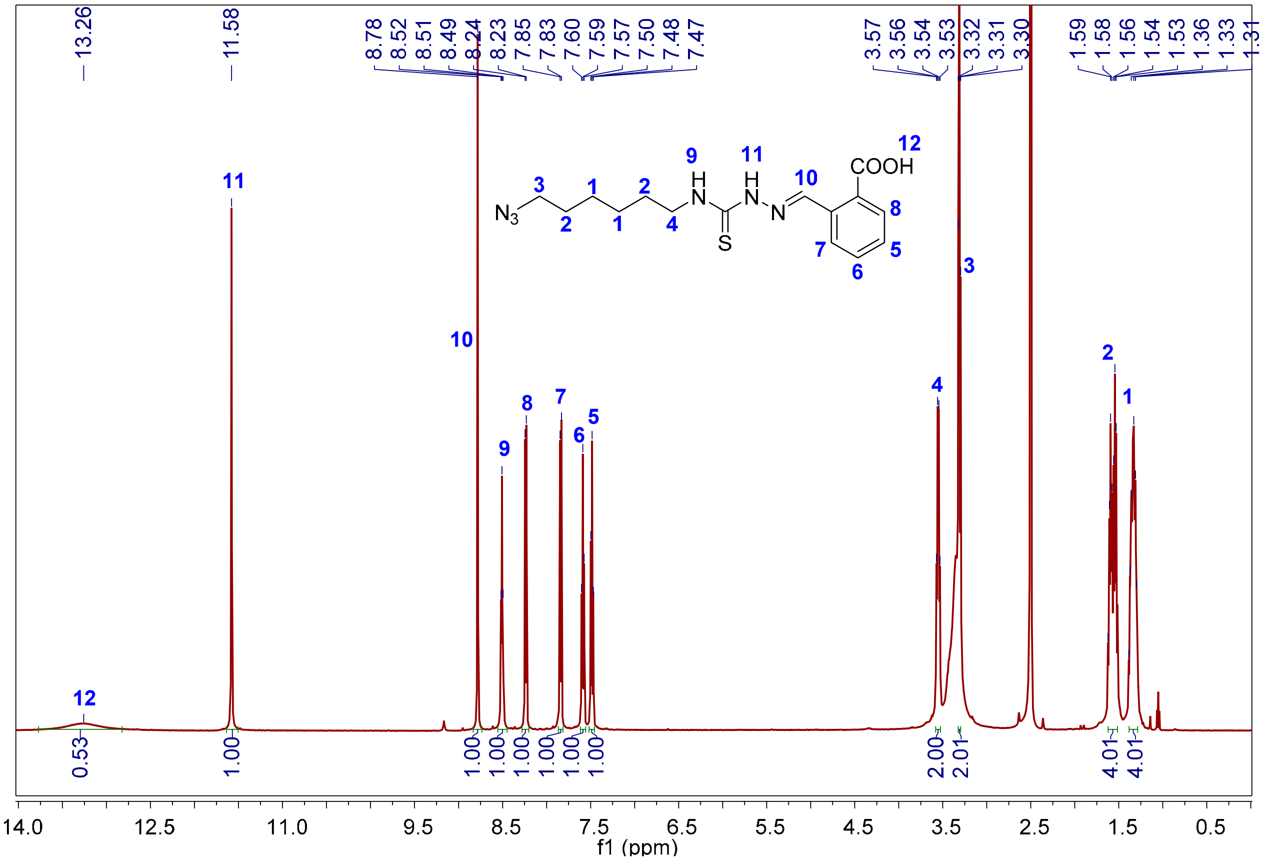

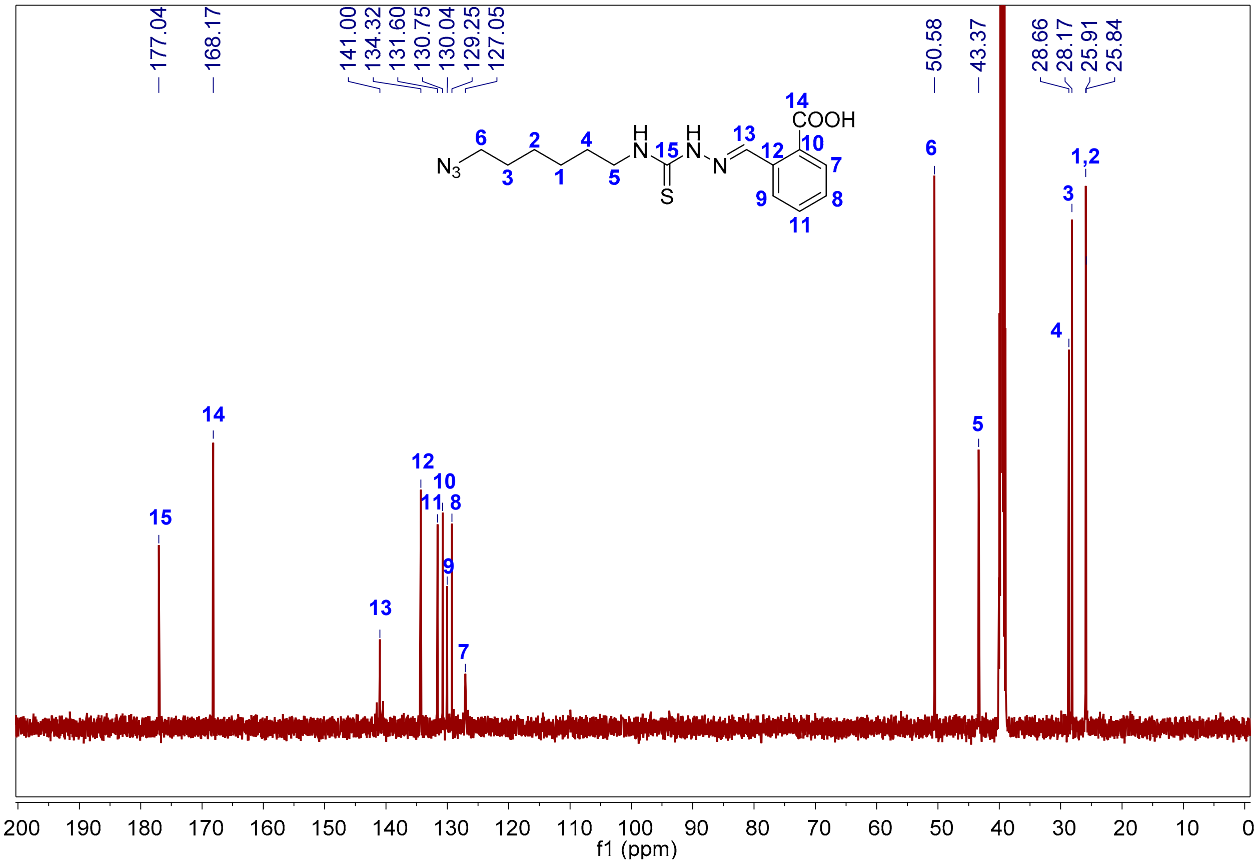

Figure S1. ^{1}H-NMR (500 MHz) and ^{13}C-NMR (125 MHz) spectra of thiosemicarbazone ligand **L** (DMSO-d_6).

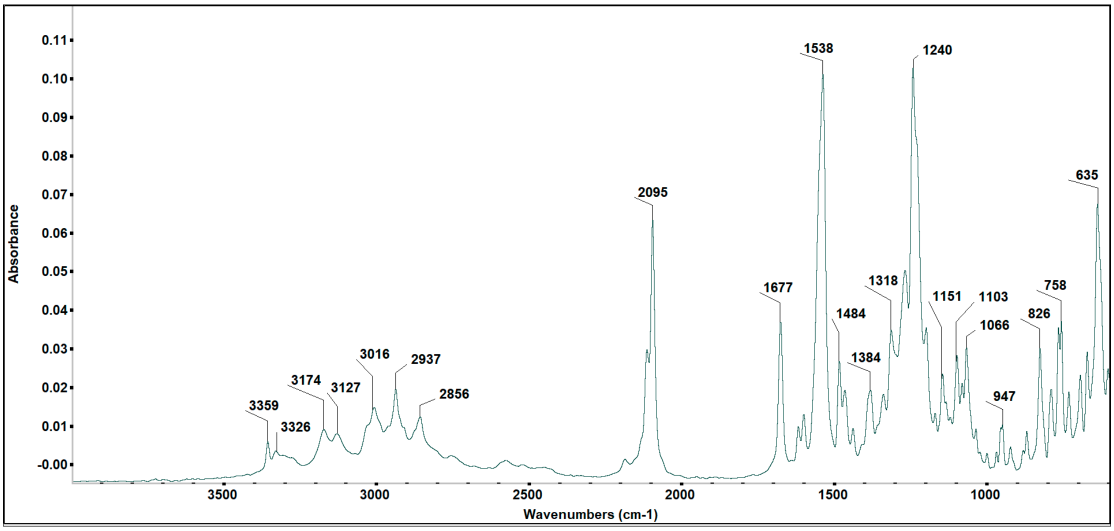

Figure S2. Infrared spectrum of thiosemicarbazone ligand **L**.

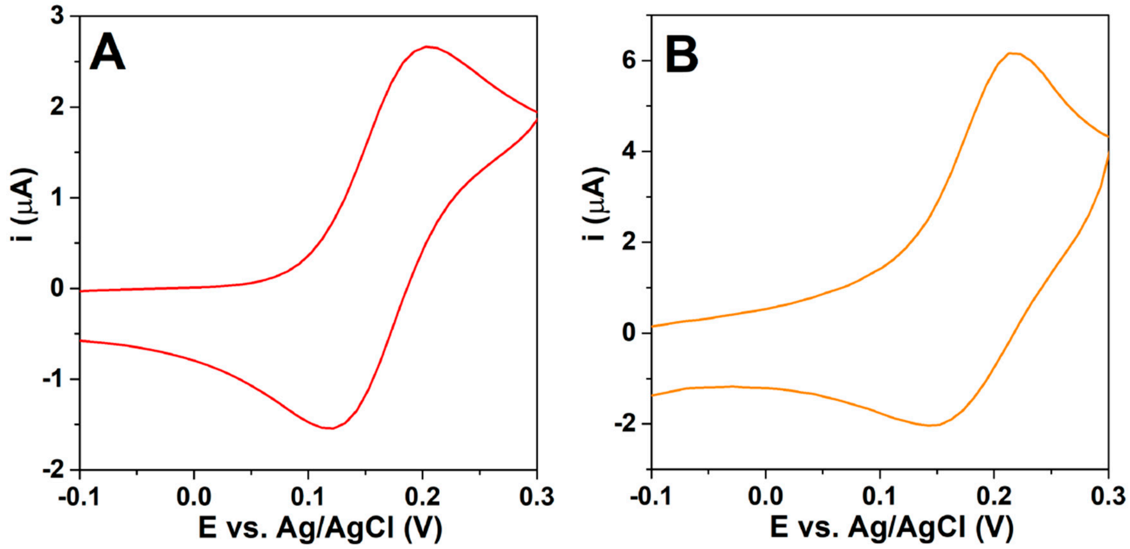

Figure S3. Cyclic voltammograms recorded in 1 mM ferrocenemethanol, 0.1 M KCl solution (20 mVs^{-1}), on (A) GC and (B) GC-ERGO electrodes.

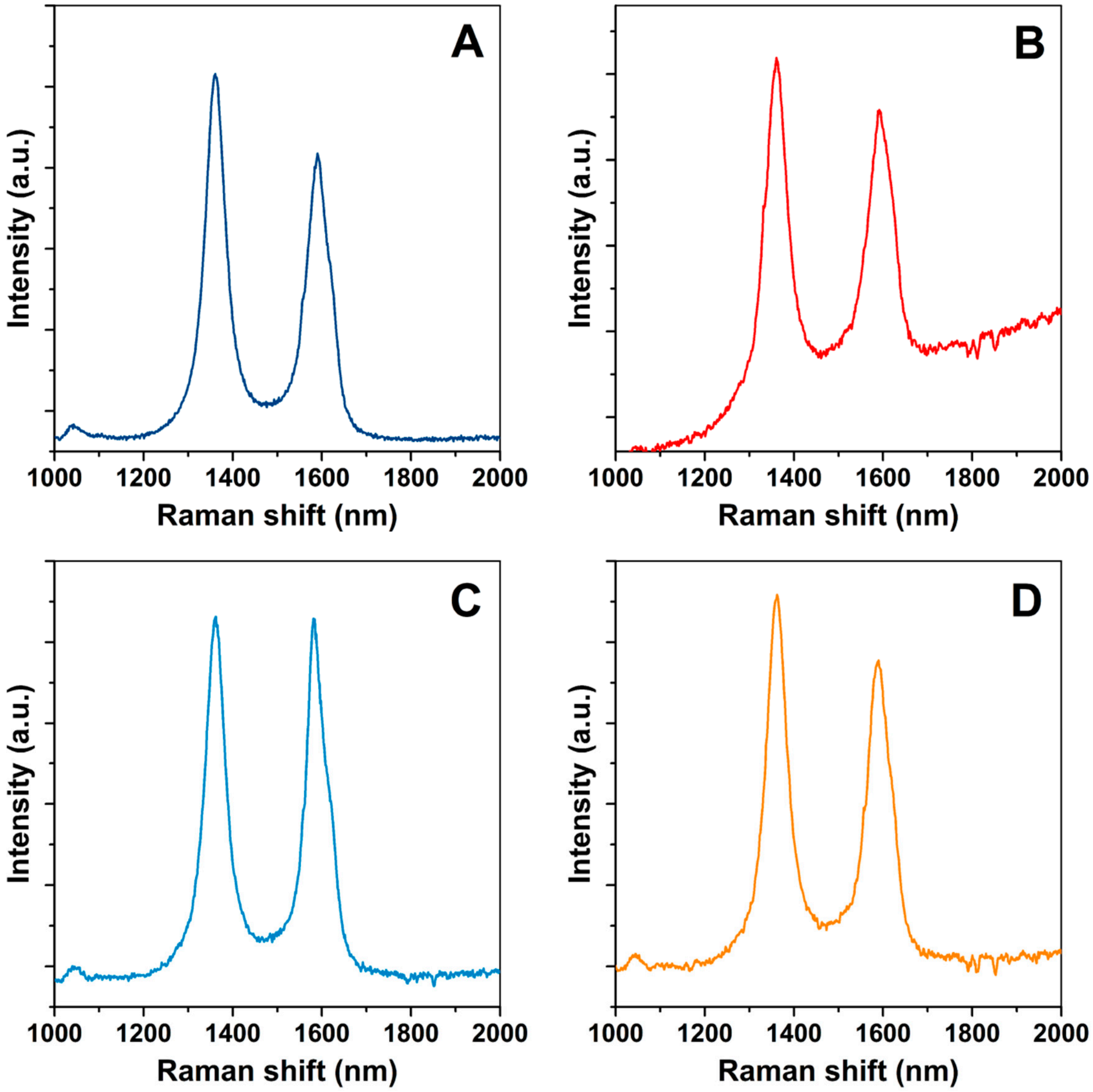

Figure S4. Raman spectra of (A) GC, (B) GC|click|**L**,(C) GC-ERGO, and (D) GC-ERGO|click|**L** electrodes.

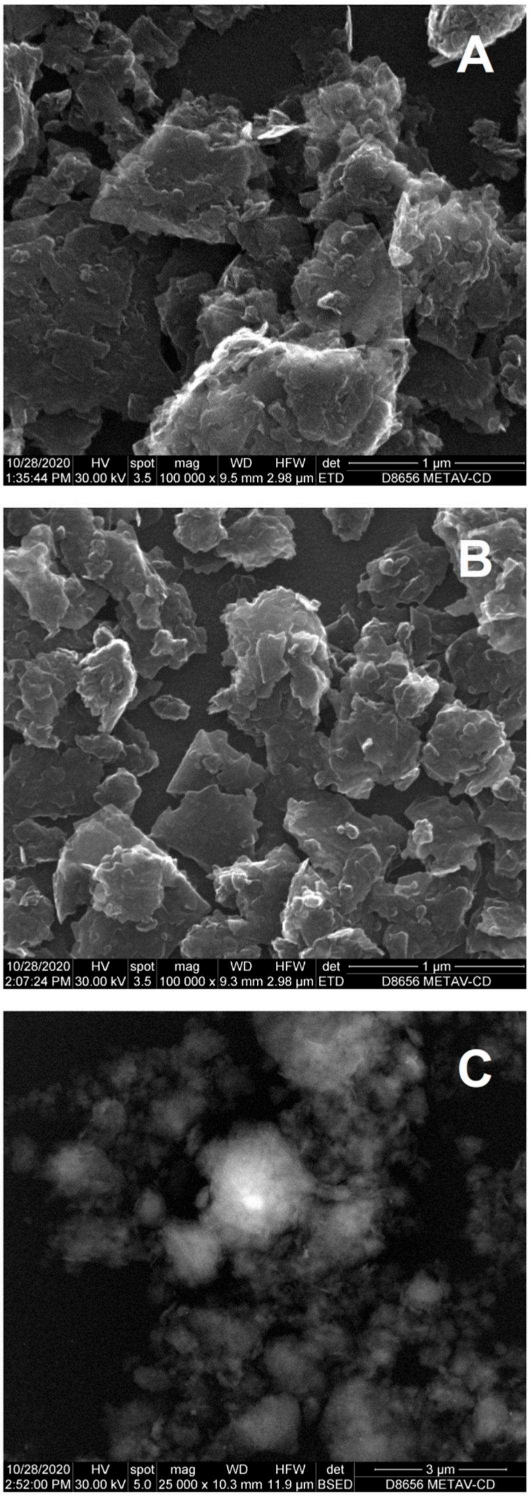

Figure S5. SEM micrographs of the electrode surfaces for A) GC-ERGO, B) GC-ERGO|click|**L**, C) GC-ERGO|click|**L** treated in a Hg(II) solution (backscattered electron detector)

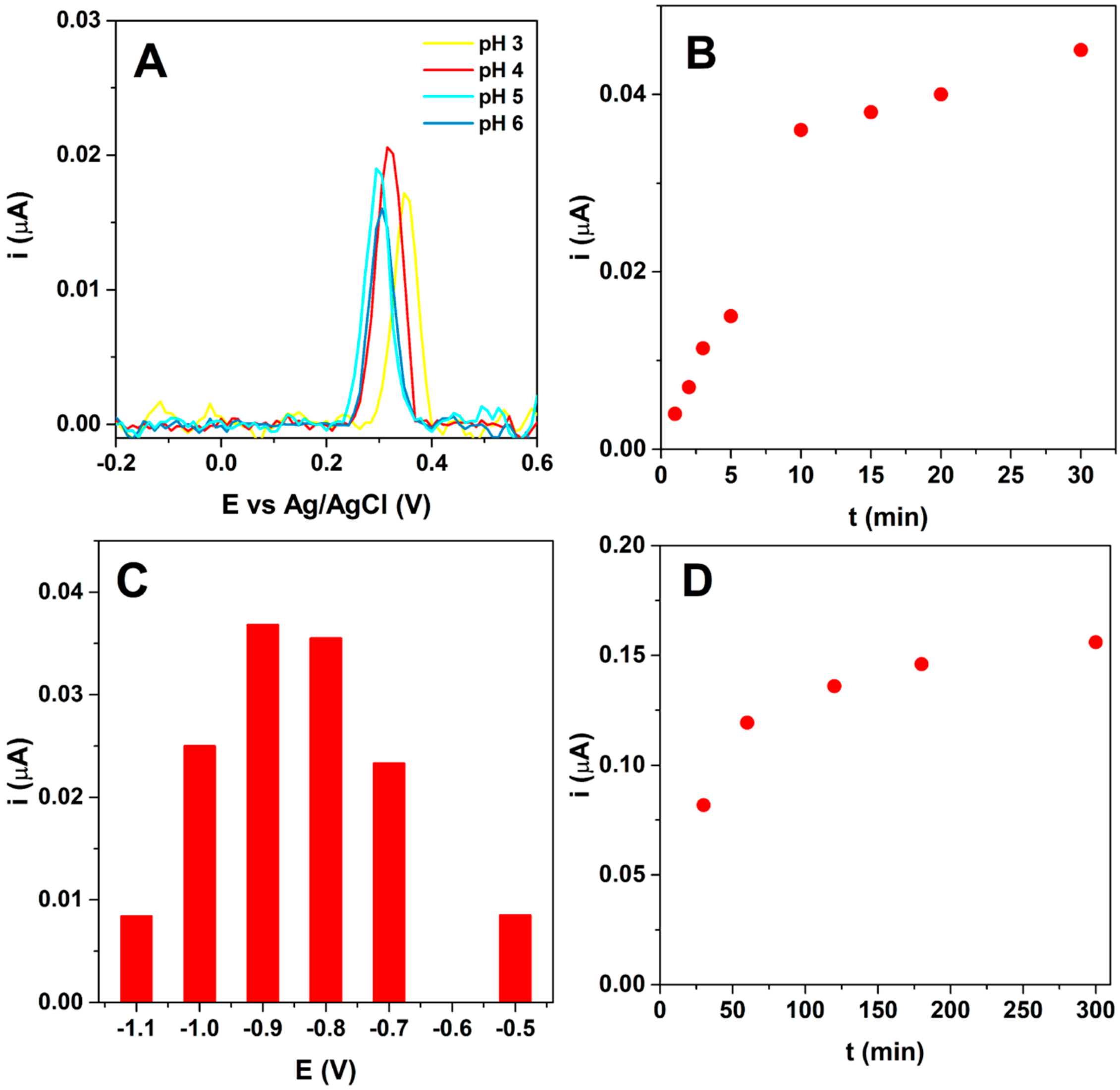

Figure S6. The effect of (A) pH of the accumulation solution, (B) accumulation time at open circuit, (C) deposition potential, and (D) time for the reduction of accumulated ions, on the Hg(II) stripping current recorded with a GC|click|**L** modified electrode.

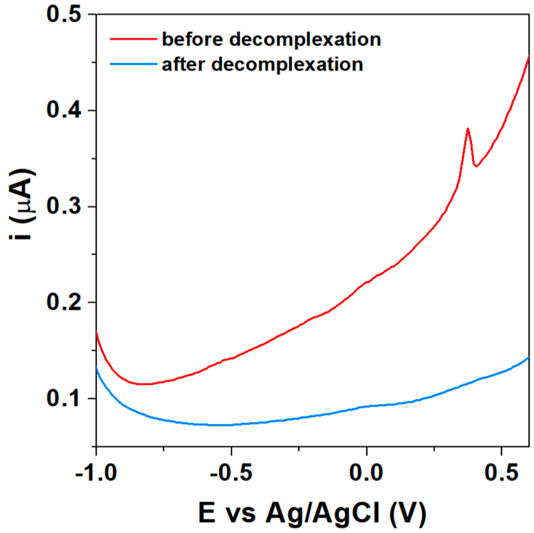

Figure S7. Hg(II) DPV curves recorded on GC|click|**L** electrodes before and after decomplexation in 1 mM sodium diethyldithiocarbamate solution.
